# Pre-hospital stroke recognition in a UK centralised stroke system: a qualitative evaluation of current practice

**DOI:** 10.29045/14784726.2019.06.4.1.31

**Published:** 2019-06-01

**Authors:** Lisa Brunton, Ruth Boaden, Sarah Knowles, Christopher Ashton, Adrian R. Parry-Jones

**Affiliations:** The University of Manchester; The University of Manchester; The University of Manchester; Salford Royal NHS Foundation Trust; The University of Manchester; Salford Royal NHS Foundation Trust

**Keywords:** focus groups, paramedic, stroke

## Abstract

**Background:**

A significant number of patients conveyed via ambulance to hyper acute stroke units (HASU) with suspected stroke have other diagnoses. This may delay treatment for non-stroke patients and cause burden to stroke teams. The Greater Manchester (GM) Connected Health Cities (CHC) stroke project links historical North West Ambulance Service NHS Trust (NWAS) data with Salford Royal Hospital electronic data to study stroke pathway compliance and accuracy of paramedic diagnosis and aims to use these data to improve pre-hospital clinicians’ accurate recognition of stroke through development of service improvement innovations. We report on supplementary qualitative work required to understand stroke recognition from the pre-hospital clinician’s perspective.

**Methods:**

Focus groups and semi-structured interviews were conducted with pre-hospital clinicians of various grades, working in the GM area of NWAS. Focus groups and interviews were audio recorded and transcribed verbatim. We used thematic analysis informed by normalisation process theory (NPT) to analyse the data. This theory helps us to understand how innovations are developed, implemented and sustained into healthcare practice.

**Results:**

Sixteen pre-hospital clinicians took part in two focus groups, one dyad interview and five one-to-one interviews. Analysis identified that respondents were unaware of false positive stroke rates entering onto the stroke pathway. Pre-hospital clinicians receive limited feedback from jobs and this impedes their ability to learn from their experiences. Respondents reported difficulty in ruling out stroke in certain patient cohorts and difficulty in recognising differential diagnoses. They expressed a lack of confidence to rule out stroke in the pre-hospital setting. They also expressed greater concern for ‘missed strokes’.

**Conclusion:**

The qualitative findings support the development of innovations to improve accurate recognition of stroke in the pre-hospital setting.

An enhanced FAST tool, better relations with HASU clinicians, feedback and education on the stroke pathway and differential diagnoses were all considered useful to improve accurate stroke recognition.

## Introduction

Stroke is the fourth largest cause of death in the United Kingdom and approximately 65% of stroke survivors live with some form of disability (Stroke Association, 2018). People with a suspected acute stroke should be admitted directly to a hyper acute stroke unit (HASU) for assessment and treatment by a specialist stroke team (Intercollegiate Stroke Working Party, 2016). Pre-hospital clinicians often provide the first point of healthcare for stroke patients (Fothergill, Williams, Edwards, Russell, & Gompertz, 2013) and national guidelines advise them to use a recognised screening tool, such as FAST (Harbison et al., 2003) to assist in the accurate and early recognition of stroke, so they can provide an appropriate emergency response (Intercollegiate Stroke Working Party, 2016; National Institute for Health and Care Excellence, 2008).

Pre-hospital clinicians need to accurately recognise stroke because taking false positive stroke patients to a HASU diverts vital resources from stroke patients and delays treatment to non-stroke patients. However, data from a one-month audit in Greater Manchester (GM) identified that up to 48% of patients arriving at GM HASUs did not have a final diagnosis of stroke (this figure included patients with a final diagnosis of transient ischaemic attack (TIA), constituting approximately 12%) (Greater Manchester Stroke ODN, 2016).

One promising way to address this problem is through better use of electronic health data to study stroke pathway compliance and the accuracy of pre-hospital clinician diagnosis. Connected Health Cities (CHC) is a government-funded initiative to improve services for patients in the north of England by creating Learning Health Systems (LHSs) (Connected Health Cities, 2018). LHSs enable improvements in healthcare through the trusted use of routinely collected electronic health data and technology (Friedman, Wong, & Blumenthal, 2010). The GM CHC stroke project aims to improve pre-hospital clinicians’ accurate recognition of stroke by linking historical North West Ambulance Service NHS Trust (NWAS) data with Salford Royal Hospital electronic data to analyse accuracy and compliance. The GM CHC stroke project has identified that there were 4216 ambulance conveyances on the stroke pathway to Salford Royal Hospital over a period of 18 months (August 2015–February 2017). Of these, 2213 (52.5%) were stroke cases, 492 (11.7%) were TIA cases and the rest were false positive stroke cases (n = 1511 [35.8%]). The five most common non-stroke diagnoses were epilepsy (n = 244 [5.8%]), migraine (n = 241 [5.7%]), sepsis (n = 218 [5.2%]), Bell’s palsy (n = 80 [1.9%]) and syncope (n = 79 [1.9%]) (Brunton et al., 2018). As part of the CHC stroke project, we are conducting qualitative work to analyse the processes reflected in the quantitative dataset. The information gathered from the CHC stroke project will then be used to support service changes and develop enhanced stroke recognition tools for testing in GM.

While previous studies have provided some insight into pre-hospital clinicians’ views of accurate stroke recognition (e.g. see Hodell et al., 2016; McClelland, Flynn, Rodgers, & Price, 2017), the aim of our qualitative work was to:

- understand pre-hospital clinician decision making when treating a suspected stroke patient;
- explore their perceptions on how stroke recognition can be improved, and challenges to this; and
- consider how better use of electronic health data can support improvements in identification and decision making.

## Methods

This qualitative evaluation adopted a thematic approach informed by normalisation process theory (NPT) (May & Finch, 2009). Focus groups and semi-structured interviews were conducted with pre-hospital clinicians working in the GM area of NWAS. While focus groups enable social interaction and discussion between group members (Lehoux, Poland, & Daudeline, 2006), we did not want to limit participation to those only able to take part in groups; hence, semi-structured interviews allowed individuals to participate.

### Setting

In GM, stroke services are centralised: three HASUs provide acute stroke care to the population. HASUs are based at Salford Royal Hospital (open 24 hours) and Fairfield General and Stepping Hill Hospitals (both open between 06.45 and 22.45 daily).

**Figure 1:**
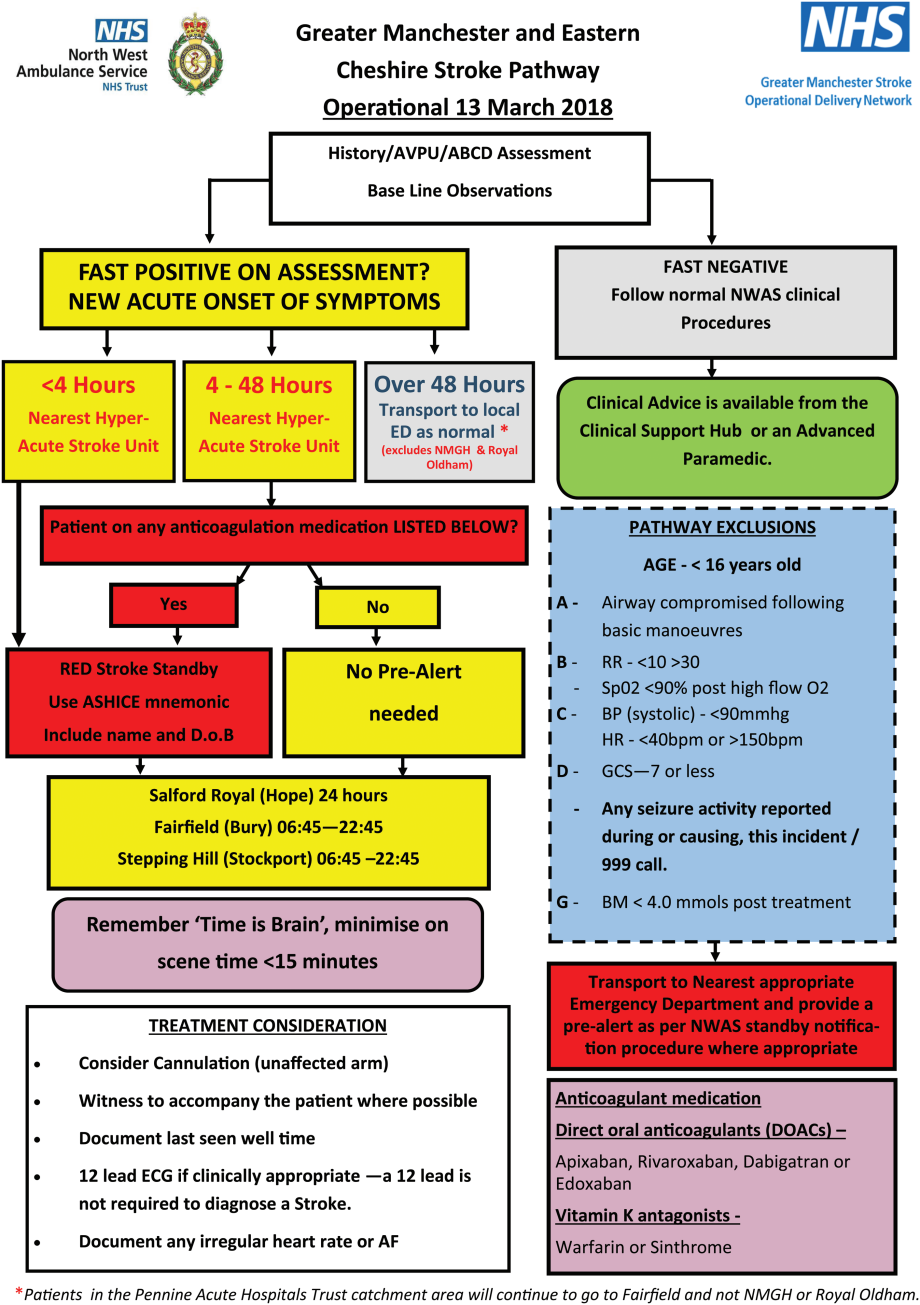
Figure 1. The GM and Eastern Cheshire stroke pathway.

Source: Reproduced with permission of Greater Manchester Stroke Operational Delivery Network.

NWAS is the second largest ambulance trust in England, providing services to a population of around 7 million people, and covers four regions: Cumbria, Lancashire, Greater Manchester, and Cheshire and Merseyside (North West Ambulance Service NHS Trust, 2019). When working in the GM region, NWAS staff follow the GM and Eastern Cheshire stroke pathway (as outlined in Figure 1) for suspected stroke patients; patients should be conveyed to the nearest open HASU unless they meet one of the exclusions, whereby they should be taken to their nearest emergency department (ED). Exclusions were created as a means to divert unstable patients to the nearest ED for safety reasons.

### Recruitment and sample

To gain a wide range of views, we aimed to recruit a varied sample and employed a mixture of convenience and snowball sampling. We aimed to conduct up to a maximum of four focus groups and aimed for a sample size of between five to eight respondents in each focus group. We advertised focus groups via posters in ambulance stations, online news bulletins and emails to team leaders. Senior paramedics (team leaders) helped to identify suitable respondents for focus groups.

### Data collection

Data were collected by LB, an experienced qualitative researcher, between August and December 2017. Data were digitally audio recorded and transcribed verbatim. Field notes were written immediately after data collection to provide context to the transcripts (Mason, 2002).

### Data analysis

NPT was adopted for the analysis as it is ideally suited in helping to understand how novel innovations can be successfully integrated into healthcare settings (Murray et al., 2010), through four overarching constructs:

- Coherence: can stakeholders make sense of the innovation?
- Cognitive participation: can stakeholders get others involved?
- Collective action: what is required to get the innovation to work in practice?
- Reflexive monitoring: can the innovation be monitored and evaluated?

NPT is most often used to evaluate innovations as they are being, or have been, implemented into practice (McEvoy et al., 2014), given its focus on embedding change into ongoing practice and processes; however, we intended to use it to inform decisions prior to implementation of any service changes. Therefore, we utilised the first two constructs of NPT (coherence and cognitive participation) in the analysis to conceptually explore stakeholder perspectives in detail prior to design. Preliminary open coding was undertaken so as not to restrict analysis to the constructs. The study analysis team (LB, SK, RB) then analysed the themes in terms of the constructs to determine applicability. It was agreed that the constructs effectively summarised attitudes towards the intervention and identified potential barriers to implementation. Further methodological details are reported in Supplementary 1.

### Ethical considerations

The evaluation was reviewed and given ethical approval from the University of Manchester (Ref: 2017-2378-3397). We gained R&D approval from NWAS (Ref: NWAS 2017_2018 161). HRA approval was not required as the evaluation did not meet their definition of research. Respondents were provided with written information before taking part and advised that participation was voluntary. All respondents signed a consent form before participating. Transcribed data were anonymised to remove any traceable information, to ensure respondents’ confidentiality.

## Results

### Respondents’ professional roles

Sixteen pre-hospital clinicians took part in two focus groups, one dyad interview (an interview conducted with two respondents) and five semi-structured one-to-one interviews (focus groups/interviews lasted for a mean average of 72 minutes). Table 1 outlines respondents’ characteristics. The majority of respondents (n = 14) worked full-time hours in 12-hour shift patterns and all worked a mix of day and night shifts. Respondents were based at different locations across the GM area, which spanned the three HASU locations.

**Table 1.** Respondents’ characteristics.

Project code	Professional role	No. years’ experience	Data collection
R01	Senior paramedic	> 10	Interview
R02	Senior paramedic	> 10	Focus group 1
R03	Emergency medical technician (EMT) 1	2–5	Focus group 1
R04	Paramedic	6–10	Focus groups 1&2
R05	Newly qualified paramedic	< 1	Focus group 1
R06	Paramedic	6–10	Focus group 1
R07	EMT 2	> 10	Dyad interview
R08	Paramedic	6–10	Dyad interview
R09	EMT 1	2–5	Interview
R10	Student paramedic/EMT 1	2–5	Interview
R11	EMT 1	2–5	Interview
R12	EMT 1	2–5	Interview
R13	Paramedic	> 10	Focus group 2
R14	Senior paramedic	> 10	Focus group 2
R15	Paramedic	> 10	Focus group 2
R16	Paramedic	> 10	Focus group 2

### Respondents’ work practices

Depending on their professional grade, respondents worked as the sole clinician in a rapid response vehicle and/or as part of a two-person ambulance crew. Respondents described how, depending on staffing levels, the skill mix in two-person ambulance crews ranged from double emergency medical technician (EMT) crews to one EMT and one paramedic:

Ideally, it should be a paramedic on every vehicle and that’ll be with a technician. We do strive for that. And most of the time we, around here, we do get that. There are occasions . . . where there’s lots of double technician crews [. . .] who obviously don’t . . . necessarily [have] the knowledge and experience. (R01, interview)

Because NWAS is a large organisation covering the North West of England, respondents sometimes worked in areas that they were not familiar with. This brought challenges if crews were not familiar with clinical pathways in areas they did not usually work in:

R07: As you finish at the hospital, your vehicle’s got a tracker on it, so if there’s an emergency nearby it’ll pick you up as being available.
R08: I think likewise not so far but being in [area] we get dragged up into [Lancashire] quite a lot . . . so we can end up in Burnley and Blackburn and sometimes as far up as Preston. With being one NWAS, one service. (Dyad interview)

### Coherence: pre-hospital clinicians’ understanding of the problem

The themes identified under ‘coherence’ relate to what extent pre-hospital clinicians felt that accurate recognition of stroke was a problem that needed to be addressed. Prior to taking part in the project, no respondents were aware of the false positive stroke rates being conveyed to GM HASUs. Lack of feedback from jobs was stated as a reason for lack of awareness:

R04: We don’t get told the figures, how many are false. I’m certainly not aware of those figures.
R02: I haven’t a clue.
R03: According to everyone I take in, I’ve ticked a pathway, they’re having a stroke.
R06: Yeah, and there’s only certain ones that you find out, like if you specifically go back. (Focus group 1)

Furthermore, while pre-hospital clinicians identified a need for more accurate stroke recognition in the pre-hospital setting, greater concern was expressed for false negative strokes and there was a strong sense of ‘erring on the side of caution’ throughout the data. These themes are explored in more detail below.

#### Understanding and awareness of misidentification was limited by absence of feedback

No formal feedback mechanism existed for the stroke pathway, so respondents did not receive feedback if they conveyed a patient, whose final diagnosis was not stroke, to a GM HASU:

I’ve never been told off for putting someone on the stroke pathway when I shouldn’t have done. (R11, interview)

However, respondents reported formal feedback mechanisms in place for other clinical pathways and expressed positive views for these, demonstrating buy-in to the idea of learning adaptively from service data in this way:

We’ve introduced, you know, when people are having MIs and whenever we took them to a [catheter] lab straightaway, we got the direct feedback [. . .] So, certainly for me . . . when that started . . . we got to know what was right. (R13, focus group 2)

Further support came from the perception that such feedback would provide opportunities for professional development:

In terms of developing as a clinician, you know, a vital part of any learning is the feedback loop, isn’t it, so you don’t know if you’re doing something particularly well that you could share with others, or if you’re repeatedly missing the same thing, if you don’t get that feedback, you’re just going to carry on making that same mistake again. (R15, focus group 2)

There was evidence throughout the data that pre-hospital clinicians strive to develop their own informal feedback mechanisms with hospital and/or primary care staff. However, respondents expressed difficulty in receiving informal feedback because, due to their working patterns and the geography of the GM area, they did not always return to the same hospital within the same shift:

But that’s another issue, we don’t tend to find out what happens. [. . .] And you can sometimes not end up going back to that hospital, just because of the geography of the area. (R10, interview)

#### Risk perceptions regarding misidentification

During focus groups/interviews, when respondents became aware of the false positive stroke rates attending GM HASUs, they identified a need for more accurate stroke recognition in the pre-hospital setting. However, throughout the data, respondents generally expressed greater concern for false negative strokes or ‘missed’ strokes. Since stroke services have been centralised in GM, respondents felt that pre-hospital clinicians were more likely to place patients on the stroke pathway to ‘err on the side of caution’ because they felt that incorrect conveyance of an acute stroke patient to a district general hospital may result in suboptimal care. This was linked to pre-hospital clinicians’ awareness that acute stroke requires time-sensitive treatment. Respondents stated that they preferred to be in the ‘right place’ and be wrong about the patient having a stroke, than risk taking a patient experiencing a stroke to the ‘wrong place’ as the effects of not treating a stroke in time could be devastating for the patient and their family:

[If we] take them to a different A&E and then it is really a stroke and their life is badly affected because of the [stroke] . . . because it isn’t just them that’s affected, it’s the carers, it’s the family. Everyone gets affected by stroke. It’s, it’s a horrible, horrible, horrible thing to happen to anyone. (R09, interview)

Furthermore, conveying false positive stroke patients to a HASU was not perceived by respondents to be detrimental to patient care. Part of this is due to the geography and structure of GM HASUs, whereby respondents felt justified in bypassing local EDs to convey patients to a HASU as the difference in distance/time between the two was often ‘insignificant’:

R15: You know, if the stroke unit was on a hill, 10 miles from any hospital, and you had to make a decision . . .
[. . .]
R04: They’re still going to get treated for whatever it is.
R15: They’re in A&E with the best neuro we’ve got.
R14: . . . I completely agree with you, the extended journey time to come to [HASU] as opposed to [local A&Es] is that insignificant that you feel if there’s any doubt, they’re going to an A&E with [neuro and HASU] . . . it’s a safer call for the patient, isn’t it? (Focus group 2)

They also considered that if the diagnosis turned out not to be a stroke, patients were still in a suitable place (i.e. a fully equipped ED where they could be referred on to other specialities). Erring on the side of caution was also linked to respondents’ perception that they were working within a ‘risk averse’ organisation:

R08: NWAS is such a risk averse service that for them, for them to introduce something that could increase the possibility of us misdiagnosing, I don’t think they’d touch it. I don’t think they’d go anywhere near [it] . . . And I think that’s why it comes down to [using] FAST . . . as a tool . . . is that anybody who’s FAST positive goes [to HASU] regardless of whether you think it’s a stroke or not.
R07: And that way you’re avoiding all risk of missing a stroke. (Dyad interview)

### Cognitive participation: pre-hospital clinicians’ perception of role in stroke recognition

Analysis using the cognitive participation construct enabled us to consider whether respondents felt that they were the right people to be involved in interventions to improve accurate stroke recognition, and how current procedures impacted their role and relationships with other professionals. Some respondents perceived false stroke rates to be ‘inevitable’ in their role, suggesting they did not always have the skills or tools within the pre-hospital setting to differentiate a false positive stroke from a ‘true’ stroke:

. . . us on the road, we’ve got to take everything at . . . pretty much at face value, and just go with the flow, so to speak. Um, and bar having a scanner, I don’t think . . . I don’t think there is anything, really [to reduce false positive strokes entering the stroke pathway]. (R11, interview)

However, respondents were eager to develop the pre-hospital clinician role and keen to improve their stroke recognition. In addressing this issue, respondents identified changes (i.e. interventions) they felt were required to improve stroke recognition in the pre-hospital setting. These sub-themes are explored in more detail below.

#### Difficulty ruling out stroke in the pre-hospital setting

Respondents expressed difficulties in ruling out stroke and making differential diagnoses in the pre-hospital setting. In discussing the difficulty of recognising differential diagnoses, there was a sense that pre-hospital clinicians lack knowledge and confidence to rule out stroke:

R06: I’m a reasonably confident clinician but that’s definitely something . . . where I go, if someone presented with like a hemiplegic migraine, and that gives you weakness and tingling, numbness and visual disturbance, again I’m going to go on the stroke pathway.
R04: Yeah, I would yeah. I totally agree with that, yeah. (Focus group 1)

This stemmed, in part, from a perceived lack of training in clinical pathways and differential diagnoses:

Historically if [NWAS] put a pathway in [it’s] ‘there’s your pathway’, there’s nothing to say why that pathway is like that, the decision-making behind why . . . say if we say ‘if they’re pyrexic, they’re excluded’ [. . .] And there’s no training, and [. . .] if you interpret that wrong . . . (R15, focus group 2)

The FAST test was reported as problematic to use with certain cohorts of patients, for example when attending patients who lived in care homes. Often these patients had pre-existing conditions which resulted in a facial droop or one-sided weakness and care home staff were not always familiar enough with the patients’ condition to recognise if symptoms had worsened or not. Some respondents described the difficulty of carrying out the FAST test with certain cohorts of patients, for example those who had dementia or those who were intoxicated with alcohol, making accurate recognition of stroke more difficult:

R04: In a severely disabled person, it’s quite limited.
R13: Or someone with dementia, or someone who’s got . . .
R16: Someone with a known weakness for some other reason.
R13: Or an ongoing infection or something that can’t take direction.
R15: If they’re agitated for a reason.
R04: Somebody that’s had a previous [stroke] and has got some weakness anyway from that previous event. (Focus group 2)

They also described conditions which ‘mimicked’ stroke symptoms, which included conditions such as migraine, Bell’s palsy and infection in elderly patients. In addition, respondents felt constrained by the use of the FAST tool, because it did not allow them to use their ‘clinical judgement’ to rule out stroke:

R04: It’s quite a rigid pathway isn’t it? Do you know what I mean, and if you tick those boxes?
R06: That’s it, they’re going. And it’s like, it’s out of your hands then.
R04: Yeah. There’s not much scope for clinical judgement really. (Focus group 1)

Respondents reported several cases where they felt they had ‘no option’ but to put patients on the stroke pathway, even when their clinical instinct was to think that the patient was not experiencing a stroke.

#### Feedback and learning

In recognising the need to improve stroke recognition by pre-hospital clinicians, respondents described a number of changes required. There was a tension in the data between respondents who articulated concern about using the same tool to assess stroke as the general public, and others (mainly EMT1s) who felt that FAST was a useful, simple tool that could be used by all grades of staff:

R04: [FAST is] limited, because you’re purely using exactly the same tool that they put on an advert on telly to tell people that live in their house to assess . . . if you follow the . . .
R14: The FAST, yes.
R04: Yes, do you know what I mean? [. . .] it’s very blunt, it’s very simplistic in many ways. (Focus group 2)
I think from the point of view of an EMT, a double EMT crew, [FAST is] great because you get a definitive yes or no answer. (R12, interview)

Nevertheless, the majority of respondents identified a need to enhance the FAST test/GM stroke pathway. This included wanting to enhance their current stroke pathway to increase specificity (i.e. to more accurately identify when patients were not experiencing a stroke), but also wanting to make the pathway more sensitive to patients who may present as FAST negative but are experiencing a stroke:

R08: And it’s not just ‘okay, this person’s FAST positive so they must be having a stroke’ . . . but then as we discussed earlier, the tool means that if they’re FAST positive we have to assume that it’s a stroke. So that, that kind of . . .
R07: I do think they need to . . . they need to bring more in to, to elaborate on it. I think we’ve had FAST long enough, and it’s working, but perhaps we need to look a little bit more in-depth. (Dyad interview)

The majority of respondents would welcome the creation of a formalised feedback mechanism for stroke cases; however, to enhance professional development, they stated that feedback needed to be timely and constructive:

[Formal feedback] would be great. But it needs to be kind of within a couple of weeks, not six, seven, eight months down the line [. . .] It needs to be something fresh enough. (R09, interview)

Only one respondent, an EMT1, had concerns that receiving individual feedback could negatively influence future decisions in stroke recognition:

But, what I would be a little bit wary of is if we were getting feedback that we’d taken somebody in with symptoms X, Y and Z and in fact the diagnosis was us being . . . erring on the side of caution [. . .] I wouldn’t want to use it as a feedback loop to, to influence future decisions. (R12, interview)

There was a sense that pre-hospital clinicians felt ‘pushed aside’ by HASU staff, with respondents reporting conflict between them and HASU staff:

A lot of conditions, we got a good idea of what’s going to happen when we get to [emergency department], with strokes it’s almost like ‘thank you very much, bye’. That’s the kind of attitudes you get from a lot of stroke teams: ‘thank you; see you later. This is our bubble’. (R02, focus group 1)

Respondents expressed a need for greater communication between pre-hospital clinicians and HASU clinicians, which they felt would help them to gain a better understanding of each other’s roles and lead to better training opportunities. Furthermore, respondents emphasised that any service changes in the pre-hospital setting needed to be supported by hospital clinicians in the secondary care setting:

Very high, senior things, including funding and commissioning, will be done and then [that is] usurped by local triage nurses or what have you . . . the reaction of local triage nurses and doctors [can] bend the practice of local crews. (R15, focus group 2)

Suggestions for improving stroke training included student paramedic placements on HASU, organising workshops facilitated by hospital stroke specialists and better utilisation of NWAS’s online training platform. Respondents suggested ways in which they could learn from service data; for example, by presenting ‘case studies’ of false positive and false negative stroke cases in NWAS’s clinical bulletins:

R16: But even just case studies on that, you know? Particularly if there are patterns of ones that we’re all failing or certain people are failing on, or misdiagnosing whatever, if you could do a case study discussion on those [. . .]
R13: [You could put it in the] clinical bulletin, couldn’t you? (Focus group 2)

## Discussion

In order to provide insight into reasons for non-compliance with the GM and Cheshire stroke pathway and conveyance of non-stroke patients to GM HASUs, we conducted a qualitative evaluation to explore pre-hospital clinicians’ perception of stroke recognition in a UK centralised stroke service.

We found that pre-hospital clinicians’ decision making when treating suspected stroke patients was influenced by their perception of risk. Pre-hospital clinicians working within a centralised stroke system expressed greater concern for misidentification of false negative strokes than false positive strokes: their decision making focused around ‘erring on the side of caution’ and this was further influenced by their perception that they worked within a risk averse organisation.

In addition, pre-hospital clinicians face challenges which impede their ability to accurately identify stroke. These include lack of feedback, difficulty in ruling out stroke in certain patient groups, use of limited tools and a perceived lack of knowledge and confidence to make differential diagnoses in the pre-hospital setting. Our findings support those reported by Hodell et al. (2016) who identified similar perceived barriers to accurate recognition of stroke among paramedics in the United States. They found that the diversity of stroke presentations, linguistic differences, patients’ alcohol or drug intake, lack of feedback from hospital staff and lack of paramedic stroke education all impeded accurate stroke recognition in the pre-hospital setting (Hodell et al., 2016).

In line with regional ambitions to develop LHSs, through use of electronic health data to support improvements in decision making and identification of stroke, we found that the majority of respondents supported an enhancement to FAST and would welcome data-driven feedback mechanisms to improve stroke recognition. This supports previous research where paramedics were supportive of using enhanced tools to improve their recognition of false positive strokes (McClelland et al., 2017). McClelland and colleagues gained 231 paramedics’ views of stroke training and practice. They found that 97% of paramedics surveyed used the FAST tool, 82% wanted more stroke training and 65% were agreeable to a tool to predict false positive stroke patients in the pre-hospital setting (McClelland et al., 2017).

Data-driven learning initiatives, and LHSs more broadly, can be described as ‘sociotechnical’ systems (Friedman et al., 2010). The importance of supplementing ‘big data’ (i.e. quantitative data) with ‘deep data’ (i.e. qualitative data) when developing data-driven innovations is well recognised (Atkins, Kilbourne, & Shulkin, 2017). Our qualitative evaluation has identified that, in developing innovations to improve stroke recognition in the pre-hospital setting, it is crucial to consider pre-hospital clinicians’ existing work practices and the organisational culture in which they work; using NPT as a guiding analytical framework has enabled our understanding of this.

### Limitations

The evaluation involved a small sample of pre-hospital clinicians who worked within a metropolitan area with a centralised stroke service; therefore, the findings are unlikely to be transferable to other areas of the United Kingdom with different stroke service models. However, evidence from London and GM (which shows that centralisation of stroke services can lead to a reduction in mortality and length of acute bed stay) is encouraging several areas in the United Kingdom to consider reconfiguring stroke services to a centralised model (Morris et al., 2019). Therefore, these findings may have implications for such areas.

It is also important to recognise the specific geography of GM. As identified in the results section, respondents did not perceive conveying false positive stroke patients to a HASU to be detrimental to patient care, because: 1) the difference in conveyance time between the local ED and the nearest HASU was considered insignificant; and 2) ED was perceived to have the capability to refer non-stroke patients to other specialities. Findings may differ in rural areas where ED by-pass transfer times are likely to be longer or in areas where HASUs are not co-located within a facility that also offers an ED.

In addition, while our findings support McClelland et al.’s (2017) findings that the majority of pre-hospital clinicians want more stroke training, our sample was made up of respondents who (in the main) had more than five years’ experience as a pre-hospital clinician. There is a core curriculum for paramedic education (College of Paramedics, 2017) in the United Kingdom, and in more recent years this has covered in-depth stroke training for paramedic students in the GM area (Greater Manchester Stroke ODN, 2017). Hence, student paramedics currently being trained in GM may have a deeper depth of knowledge about stroke assessment and differential diagnoses and this might alter their decision making from the respondents we sampled. Furthermore, delivery of paramedic education in stroke as part of the core curriculum may differ across UK higher education institutions and across different ambulance trusts, leading to a difference in pre-hospital clinicians’ decision making.

Finally, in the main, respondents were opportunistically sampled; therefore, they were motivated to take part in the evaluation and may have expressed greater motivation for their role than other pre-hospital clinicians. These limitations need to be considered when interpreting the findings.

## Conclusion

This qualitative evaluation has explored pre-hospital stroke recognition in a centralised stroke system in GM, from the pre-hospital clinicians’ perspective. We explored pre-hospital decision making when treating suspected stroke patients and identified views on how stroke recognition can be improved in the pre-hospital setting, including their perception on how data can support improvements in identification and decision making. Findings collected from the GM CHC project (both quantitative and qualitative data) will be used to support the development of innovations to improve accurate stroke recognition by pre-hospital clinicians across GM and beyond.

## Conflict of interest

None declared.

## Funding

This project is funded by the Department of Health and Social Care (Greater Manchester Connected Health City). The views expressed are those of the authors and not necessarily those of Greater Manchester Connected Health City or the Department of Health and Social Care.
